# The feasibility of harmonizing gluten ELISA measurements

**DOI:** 10.1016/j.foodchem.2017.04.092

**Published:** 2017-11-01

**Authors:** Malgorzata Rzychon, Marcel Brohée, Fernando Cordeiro, Reka Haraszi, Franz Ulberth, Gavin O'Connor

**Affiliations:** European Commission, Joint Research Centre, Institute for Reference Materials and Measurements, Retieseweg 111, Geel 2440, Belgium

**Keywords:** Gluten, Wheat, Food allergen, Celiac disease, ELISA, Gluten-free, Reference material, Commutability

## Abstract

Many publications have highlighted that routine ELISA methods do not give rise to equivalent gluten content measurement results. In this study, we assess this variation between results and its likely impact on the enforcement of the EU gluten-free legislation. This study systematically examines the feasibility of harmonizing gluten ELISA assays by the introduction of: a common extraction procedure; a common calibrator, such as a pure gluten extract and an incurred matrix material. The comparability of measurements is limited by a weak correlation between kit results caused by differences in the selectivity of the methods. This lack of correlation produces bias that cannot be corrected by using reference materials alone. The use of a common calibrator reduced the between-assay variability to some extent, but variation due to differences in selectivity of the assays was unaffected. Consensus on robust markers and their conversion to “gluten content” are required.

## Highlights

• Different ELISA kits for measuring gluten yield different responses.
• The differences between pairs of kits can vary by a factor of 20.
• The current measurement system cannot fully support legislative gluten-free claims.
• Comparability of measurements is limited by poor correlation between kit results.
• Lack of correlation creates bias that cannot be removed using reference materials.

## 1 Introduction

Gluten is defined for legislative purposes as “a protein fraction from wheat, rye, barley, oats or their crossbred varieties and derivatives thereof, to which some persons are intolerant and that is insoluble in water and 0.5 M NaCl” CODEX STAN 118-1979 (Codex Alimentarius, 1979 rev. 2008). The proteins that form gluten are major storage proteins and represent between 70% and 80% of the total protein content of grain (Scherf, Koehler, & Wieser, 2015). Consequently, gluten is found in many staple foods in the Western diet. Due to its unique functional properties, wheat gluten is also widely used in the food industry as an ingredient essential for formulating high-quality baked goods. It is also used in a range of foodstuffs other than bakery products such as meats, noodles, sausages, breakfast cereals, meat and cheese replacements and other products (Day, Augustin, Batey, & Wrigley, 2006).

Gluten is responsible for a number of immune-mediated disorders, which include celiac disease (CD), dietary wheat allergy (WA) and gluten sensitivity (GS) (Sapone et al., 2012). The spectrum of clinical manifestations includes gastrointestinal symptoms (such as abdominal pain, nausea and diarrhoea), inflammatory disease of the small intestine, which can result in nutrient malabsorption and/or allergic reactions including anaphylaxis. To date the clinically relevant thresholds for WA and GS have not been established. However, in the case of CD patients, damage to the small intestinal mucosa can be induced even when trace concentrations of gluten are present (Sapone et al., 2012). For patients suffering from gluten-related disorders, the only available treatment is the life-long elimination of gluten from their diet.

The prevalence of CD is approximately 1% in regions populated by individuals of European origin. However, due to the increase in popularity of a western style diet, which is gluten-rich, the diagnosis of CD is increasing globally (Lionetti, Gatti, Pulvirenti, & Catassi, 2015). WA affects roughly 1% of the world’s population (Scherf et al., 2015). The prevalence of GS is unknown, although estimates range from 0.6% to 6% of the population (Czaja-Bulsa, 2015).

To assist patients with gluten-related disorders in making sound dietary choices, food labelling legislation exists in many countries. These classify foods according to the gluten level they contain. The Codex Alimentarius standard defines foods as ‘gluten-free’ if the gluten level does not exceed 20 mg kg^−1^ in total and recommends the threshold of 100 mg kg^−1^ for the labelling of low gluten foodstuffs that have been specially processed to reduce their gluten content (Codex Alimentarius, 1979 rev. 2008).

The 20 mg kg^−1^ gluten free threshold has been adopted for foods bearing a ‘gluten-free’ label by the regulatory bodies in the European Union, the United States of America and Canada. However, in Australia and New Zealand a ‘gluten-free’ claim can only be made when there is no detectable gluten present. Less restrictive supplementary standards have been established in the EU at the threshold of 100 mg kg^−1^ (‘very low gluten’) and in Australia and New Zealand at 200 mg kg^−1^ (‘low-gluten’) (ANZFA, 2012, EC, 2009, FDA, 2013, Health Canada, 2012).

The global market for gluten-free products was valued at USD 3.4 billion in sales in 2014 and is expected to grow (Scherf et al., 2015).The conformity of food products with “gluten-free legislation” is most commonly assessed with immunoassays. In recent years, several enzyme-linked immunosorbent assays (ELISA) have been developed and commercialized. However, there is a wide variation in the reported measurement results between different commercially available test kits (Bruins Slot et al., 2015, Diaz-Amigo and Popping, 2012, Sharma, 2012). This variation is well illustrated by the necessity to separate the results of proficiency tests into sub-sets according to the ELISA kits used. The separate treatment of results is necessary because ELISA kits from different manufacturers form different populations and no single statistical assessment of all the results can be carried out (FAPAS, 2012, Scharf et al., 2013, Sykes et al., 2012).

The consequences of these discrepancies for the risk management of gluten ingestion are worrying. People with health conditions which require the elimination of gluten from the diet, who confidently buy food products labelled as ‘gluten-free’, are put at risk of suffering from adverse effects of gluten exposure. Market surveys aimed at assessing the rate of gluten contamination in products sold as gluten-free suggest between 1 and 20% of the products do not comply with current legislation (Lee et al., 2014, NSWFA, 2011, Sharma et al., 2015). However, these claims are derived from measurements using the very kits that are known to provide highly variable results. This may result in unnecessary actions such as market recalls or legal claims. In 2015, the EU-wide Rapid Alert System for Food and Feed received 126 allergen notifications. Twenty-three of these concerned gluten or wheat and resulted in product recalls, withdrawals or border rejections (RASFF Portal, accessed 10/12/2015). The lack of reliable monitoring of cross-contamination during food production adds to the financial burden of preventive measures which go beyond the legislative requirements. Moreover, this contributes to the excessive use of precautionary (‘may contain’) labelling which results in confusion among the consumers. A further concern is the verification of the absence of gluten cross-contamination in naturally gluten-free foodstuffs. These are commonly used in a gluten-free diet but are not necessarily labelled as ‘gluten free’.

The supposed reasons for the observed discrepancies between results of different gluten assays have been extensively discussed (Bruins Slot et al., 2015, Diaz-Amigo and Popping, 2012) and cover differences in sample preparation protocols, choice of kit buffer, antibody specificities and calibration procedures. Much focus is put on the absence of reference materials, whose availability is often perceived as the remedy to the current problems with gluten quantification (Diaz-Amigo and Popping, 2012, Sykes et al., 2012).

In this paper, we evaluate the feasibility of harmonizing gluten ELISA assays. We assess the current variation between results, both in quantitative and qualitative terms and perform correlation studies for gluten assays. The study also systematically examines the effect of: the introduction of a common extraction procedure; a common pure protein-based calibrator; and an incurred matrix reference material, on the comparability of gluten results.

## 2 Materials and methods

### 2.1 Materials

The gluten material used in this study was a commercially available, industrially extracted wheat gluten (GluVital™) batch from the year 2010 (Cargill, Bergen op Zoom, NL).

BME-IGRM-0, BME-IGRM-10 and BME-IGRM-50 are candidate reference materials produced at the Budapest University of Technology and Economics, Hungary (Bugyi et al., 2012). The materials consist of a blank baked cookie and cookies incurred with 10 mg kg^−1^ and 50 mg kg^−1^ of the WGPAT gliadin. WGPAT gliadin is an isolate from a mixture of 28 European wheat varieties prepared by the Working Group on Prolamin Analysis and Toxicity (van Eckert et al., 2006).

In the first study, we used a set of surrogate samples spiked and incurred with the industrial wheat gluten extract at 20 mg kg^−1^ and 100 mg kg^−1^. All ingredients used in their preparation, were purchased from local grocery stores.

In the second study, we used real food samples and test items from proficiency testing studies. They represented the food categories, as reported in market surveys, most commonly used by people following a gluten-free diet (Lee et al., 2014, NSWFA, 2011, Sharma et al., 2015) and contained cereals, bakery products, snacks, diet foods and food supplements, meat, confectionery and condiments.

Most of the food samples chosen fell in the 5th, lower-left sector of the food matrix organisational scheme, of the AOAC Food Triangle (Wolf & Andrews, 1995), which represents food matrices high in carbohydrates and low in fat and proteins. Three samples represented three other sectors with higher relative levels of fat and proteins (sector 4, 6 and 9).

The food samples studied were routine test samples submitted to control laboratories for gluten testing and were kindly provided by the Western Regional Public Analyst’s Laboratory (Galway, Ireland), Nuscana Biotechnika Laboratoryjna (Poznan, Poland) or were purchased from local grocery stores. The test items included T27142AQC (Cake mix); T27142BQC (Cake mix); T27109BQC (Cake Mix) and T27106B (Infant Soya Formula) from FAPAS (Food Analysis Performance Assessment Scheme, Fera Science Ltd., Sand Hutton, York, UK). Between 30 g and 500 g of each sample were homogenised in a kitchen blender, vacuum packed and stored at −20 °C until it was required for analysis.

### 2.2 Characterisation of gluten material

The total protein content and homogeneity of GluVital™ vital wheat gluten was assessed by the Dumas method. A conversion factor of 6.25 was used to convert from the determined nitrogen to total protein content (International Dairy Federation, 2006). Ten aliquots of 0.2 g were analysed with a Vario Max CN elemental analyser (Elementar Analysensysteme GmbH) using l-Glutamic acid (>99.5%, Sigma) as a reference material. The total gluten content was 79.4% with a relative standard deviation (RSD) of 0.24% indicating an adequate homogeneity for the purpose of this study.

### 2.3 Spiked flours preparation

Three blank flours consisting of soy, corn and rice were used in the study. The gluten content of each blank flour was determined to be below the limit of detection (LOD) of 3 mg kg^−1^ when measured with a Ridascreen Gliadin ELISA test kit (R-biopharm, Darmstadt, Germany).

The blank flours were spiked with 100,000 mg kg^−1^ of the vital wheat gluten. This stock was further mixed with the blank flour to yield the 100 mg kg^−1^ and 20 mg kg^−1^ gluten mixtures. The homogeneity of the spiked flours was assessed with a single ELISA test kit. Ten 1 g aliquots of the spiked flours were extracted and each extract was analysed in duplicate. For each flour the RSDs of all results were less than 10% indicating that, for the purpose of this study, the spiked flours were sufficiently homogenous with respect to gluten content.

The spiked flours were vacuum packed and stored at −20 °C until required for analysis.

### 2.4 Incurred cookie preparation

Soy and corn cookies were prepared with an incurred gluten content of 100 mg kg^−1^ and 20 mg kg^−1^ by mixing egg (75 g), sugar (200 g), butter (200 g), blank flour (400 g minus the appropriate amount of gluten flour if added) and the appropriate amount of flour that had been spiked with 1000 mg kg^−1^ of gluten.

Cookies were baked at 180 °C for 16 min, ground in a kitchen blender, vacuum packed and stored at -20 °C until required for analysis.

The gluten content of all the food ingredients used in the preparation of the cookies was below the LOD (0.3 mg kg^−1^) when measured with a Wheat Protein (Morinaga) ELISA test kit.

The homogeneity of the incurred cookies was tested with a single ELISA test kit. Ten separate 1 g aliquots were extracted and analysed in triplicate. The RSDs were less than 10% indicating that the homogeneity, with respect to gluten content of all the cookie materials, was fit for the purpose of this study.

### 2.5 Extraction with generic buffer and preparation of a universal calibrator

Vital wheat gluten, 0.25 g, was extracted with 24.75 ml of a generic extraction buffer (Akiyama, Imai, & Ebisawa, 2011) (PBS pH 7.4 containing 0.5% SDS, 2% β-mercaptoethanol) by shaking overnight at room temperature. Additionally, 0.25 g of gluten was extracted with 24.75 ml of each ELISA kit’s buffer according to the kit’s protocol. The extracts were centrifuged (4500*g*, 20 min), filtered (0.45 µm), diluted 1:10 in PBS pH 7.4 and further diluted 1:2 in PBS containing 0.2% BSA to obtain a standard solution of 500 μg mL^−1^. The standard solution was further diluted with each ELISA kit’s original buffer according to the kit’s dilution protocol. A standard calibration curve of 7 points spanning the kit’s quantification range was constructed in each kit’s respective buffer, using consecutive 1:2 dilutions.

### 2.6 ELISA measurements

Seven commercial sandwich ELISA test kits were used in the assessment of the spiked flours and real food samples: AllerTek Gluten ELISA Assay (ELISA Technologies, Inc., Gainesville, FL, USA), BioKits Gluten Assay Kit (Neogen Corporation, Lansing, MI, USA), Ridascreen Gliadin (R-biopharm, Darmstadt, Germany), Wheat Protein ELISA kit (Morinaga Inst. of Biological Science, Yokohama, Japan), AgraQuant Gluten G12 Assay (Romer Labs UK Ltd., Runcorn, Cheshire, UK), Transia Plate Prolamins (BioControl Systems, Inc. Seattle, WA, USA) and GlutenTox ELISA Sandwich (Biomedal Diagnostics, Sevilla, Spain). The homogeneity of the spiked and incurred materials was tested with a single test kit (sandwich Gluten-E Nutrilinia, Transia GmbH, Butzbach, Germany).

All measurements were performed by a single analyst in a single laboratory. The analytical protocols provided by each test kit manufacturer were strictly followed unless stated otherwise. Each of the flour samples incurred at the 20 mg kg^−1^ and 100 mg kg^−1^ were extracted twice and each extract was measured in triplicate on a single ELISA plate from each manufacturer. For the food samples, we used 3 independent extractions for each sample with measurements again being performed in triplicate. Each of the 3 extracts were analysed on a separate plate from each manufacturer. Results from the first run were used to optimize the dilution range that was then applied to samples run on the remaining two plates. This approach resulted in 3 valid datasets from 3 runs for most kits with the exception of Morinaga and AllerTek (2 datasets each) and Neogen (1 dataset).

### 2.7 Data analysis

For both studies only values falling between the lowest non-zero calibrator and the highest calibration standard for each test kit were used. However, for the BioKits (Neogen) in the second study all readings above the kit LOD were included in our calculations. This was beneficial from the point of view of completeness of the study and had no influence on the conclusions drawn when compared to calculations performed when the points between the LOD and LOQ were excluded.

The data analyses were performed using Microsoft Excel and Analyse-it Method Validation edition (Analyse-it Software, Leeds, UK).

The results of absorbance readings were analysed according to the kit manufacturer’s instructions. A polynomial (cubic or quadratic) regression was used in all cases. For each calibration curve, the combined uncertainty in the polynomial regression model was calculated (Jeter, 2003, Kragten, 1994).

## 3 Results and discussion

### 3.1 Current status of gluten ELISA measurements

The equivalence of gluten measurements from seven different test kits was assessed. These kits were representative of the variety present on the market in terms of antibody specificities, sample extraction protocols and calibration procedures. They include official test methods recognized by Codex Alimentarius, AOAC International, the American Association of Cereal Chemists International AACCI and authorities in Japan (Akiyama et al., 2011, Scherf and Poms, 2015). The characteristics of the test protocols are presented in Table 1.

**Table 1:** Characteristics of the ELISA test kits used. Precision data are from kit brochures (k), collaborative studies reports (l) (Halbmayr-Jech et al., 2015, Immer and Haas-Lauterbach, 2012) or are calculated based on our experiments (e). Different extraction procedures include 1 or 2 steps. RT indicates room temperature, ® – proprietary extraction buffer components undisclosed by the manufacturer, 2-ME – 2-mercaptoethanol, SDS – sodium dodecyl sulphate, PBS – phosphate buffer saline, EtOH – ethanol, mAb – monoclonal antibody, pAb – polyclonal antibody, RSD (relative standard deviation).

			Neogen	AllerTek	Agraquant	Biomedal	Rbiopharm	Transia	Morinaga
Antibody		coating	401.21 (Skeritt) mAb	401.21 (Skeritt) mAb	G12 mAb	A1 mAb	R5 mAb	R5 mAb	anti-wheat protein pAb
		detecting				G12 mAb			
		target	HMW-glutenins, ω-gliadins	HMW-glutenins, ω-gliadins	gliadins	gliadins	gliadins	gliadins	wheat protein
Quantification		range	3 – 50	5 – 80	4 – 200	1.56 – 25	5 – 80	1.56 – 25	0.78 – 50
		unit	ppm gluten	ppm gluten	ppm gluten	ng/ml gliadin	ppb gliadin	ng/ml gliadin	ng/ml wheat protein
Calibrant			Gluten(vital wheat gluten extract)	Gluten(in cooked breads)	Gluten(vital wheat gluten extract)	Gliadin	Gliadin	Gliadin	Wheat protein standard
Traceability			Kjeldhal method (N x 6.25)	–	WGPAT gliadin	–	WGPAT gliadin	–	–
Extraction.	Sample to buffer ratio		1:10	1:10	1:40	1:10	1:40	1:40	1:19
	Buffer	1st	®, 40% EtOH	®, 40% EtOH	®	®	®	®	®, 2-ME
		2nd	–	–	80% EtOH	–	40% EtOH	80% EtOH	–
	Time	1st	90 s	15 min	40 min	40 min	40 min	40 min	12 h
		2nd	–	–	1 h	–	1 h	1 h	–
	Temp	1st	RT	45 °C	50 °C	50 °C	50 °C	50 °C	RT
		2nd	–	–	RT	–	RT	RT	–
Repeatability (intra-assay) RSD_wa_%		12.5–15% (k)	–	16% (l)	–	27% (l)	15% (k)	<10% (k)	
Reproducibility (inter-assay) RSD_ba_%		–	–	32% (l)	–	37% (l)	23% (k)	<10% (k)	
Repeatability (within-run) RSD_wr%_ (e)		10%	12%	12%	7%	5%	14%	9%	
Between-run precision RSD_br_% (e)		12%	17%	7%	11%	14%	20%	11%	

In order to assess the performance of each kit the data were treated in a way that is typically used in proficiency testing (PT) schemes, handling each test kit as a single laboratory.

The data were first assessed for trueness and precision according to ISO 5725–2 (ISO., 1994). In our first study, we analysed samples that consisted of spiked blank flours and incurred cookies. For samples spiked/incurred with 20 mg kg^−1^ of gluten extract, the observed results ranged from 4 to 101 mg kg^−1^ and from 24 to 574 mg kg^−1^ at the 100 mg kg^−1^ level. The computation according to ISO 5725-2 was done separately for the 20 mg kg^−1^ and 100 mg kg^−1^ levels. The analysis indicated that one test kit (AllerTek) produced results which were markedly different from the others, at both concentrations. However, as the goal was to evaluate the current comparability of measurements by ELISA, data from all seven test kits were retained. The calculated repeatability (s_r_, within-kit) and reproducibility (s_R_, between-kit) standard deviations were s_r_ = 21%, s_R_ = 101% at the 20 mg kg^−1^ level and s_r_ = 11%, s_R_ = 102% at 100 mg kg^−1^. When the results from the outlying kit were removed from the dataset, s_r_ = 16% and s_R_ = 47% at 20 mg kg^−1^ and s_r_ = 19%, s_R_ = 41% at 100 mg kg^−1^.

In the second study, we performed a comparison of measured gluten content in food samples. Again, in many cases the test kits generated values that differed by 10–20 times but the analysis did not identify any outlying test kit (at the 95% confidence level). The average repeatability (s_r_, within-kit) standard deviation was 17% (ranging from 3% to 32% depending on the sample) and average reproducibility (s_R_, between-kit) standard deviation was 76% (varying from 46% to 124%).

The data generated by both studies were treated following the procedure recommended in ISO 13528 (ISO, 2015). The performance of each ELISA test kit was evaluated based on z scores where z = (*χ* − X)/σ: if the calculated |z| ≤ 2 the result differed from the reference value by less than 2 times the standard deviation used for proficiency assessment and was therefore considered satisfactory. The z scores were derived using the gravimetric values (first study) or robust averages (second study) for X and standard deviations (σ) set at a value of 25% of the reference value. A value of 25% in this context defines the maximum acceptable standard uncertainty and was chosen based on values reported earlier by providers of allergen PT schemes (DLA FAPAS, 2012, GbR, 2014, National Measurements Institute, & Australian Government, 2012).

**Fig. 1:**
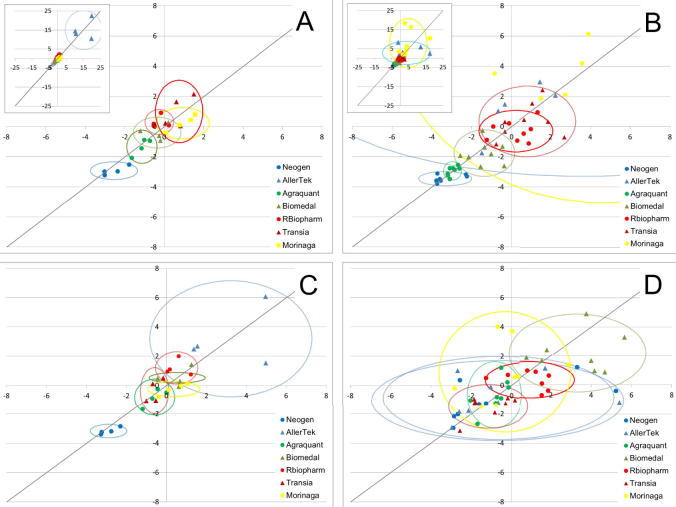
Youden plots showing the z scores derived from robust averages and standard deviation set at 25% of the reference value. The pairs of samples are matched for similar matrices. The z scores obtained for one of the samples are plotted against the z scores obtained for the other sample. Ellipses represent the mean ± 2 standard deviations for the results of each kit. A: 1st study data; B: 2nd study data; C: 1st study data corrected with universal calibrator, D: 2nd study data corrected with BME-IGRM. Two samples with largest variability in results resulting in extreme z scores (meat sample and cereals sample) were excluded from the graphs presenting 2nd study data.

In the first study, 70% of results achieved the satisfactory level of performance. Two test kits exhibited non-satisfactory performance for most of the samples. In the second study, only 43% of results achieved satisfactory performance. Youden plots presented in Fig. 1 indicate the general lack of equivalence of results among the kits. The distribution of points relative to the 45-degree reference line suggests the presence of a systematic bias: one test kit is significantly biased low in both studies (Neogen) and one test kit is significantly biased high (AllerTek). However, in the second study, this bias is less pronounced because of a presence of a higher number of extreme points in both the top right-hand and lower left-hand quadrant of the graph which represent biased results generated by other test kits (mainly the positively biased Morinaga and negatively biased Agraquant and Biomedal). The differences in the pattern of the distribution of the results between the first and second study may be attributed to a batch-to-batch variability of kits from the same manufacturer. Alternatively, they may also result from differences in responses obtained for different types of samples (depending on the matrix, level of processing etc.). In the second study, where a greater variety of sample types were used, the individual points are more scattered and ellipses enclosing the majority of results generated by individual test kits tend to overlap to a larger extent.

#### 3.1.1 Assessment of qualitative agreement between results

The lack of equivalence in gluten measurements between different ELISA kits is most problematic when placed in the context of interpreting results in respect to the legislative thresholds.

**Fig. 2:**
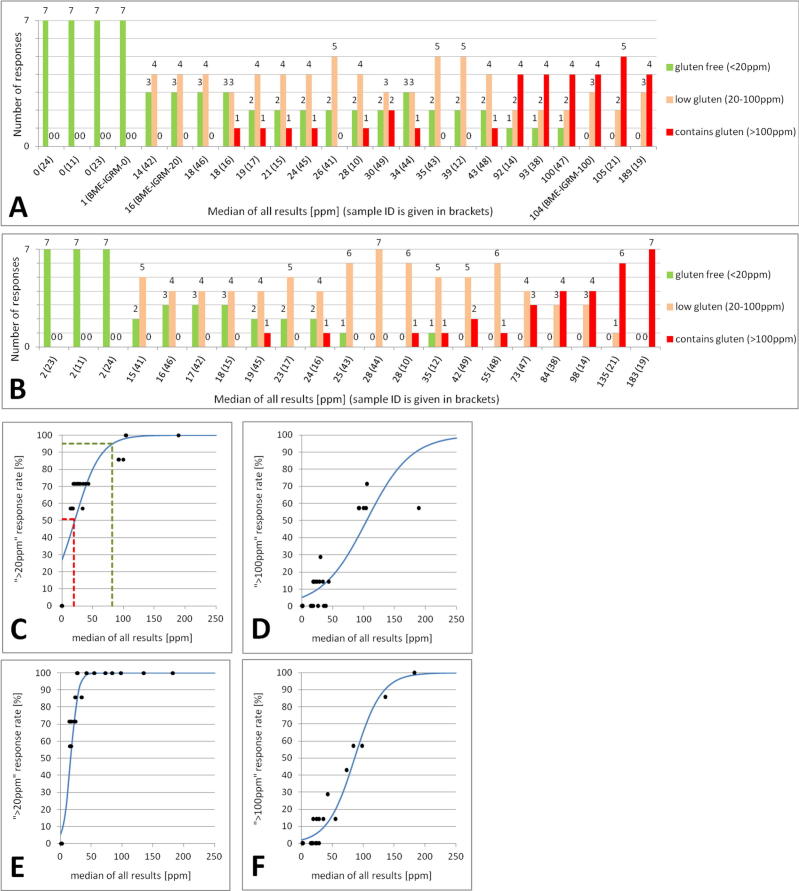
Assessment of the qualitative agreement between results before and after correction with BME-IGRM. Column plots show number of statements with regards to the gluten content (gluten-free, low gluten, contains gluten) per sample before (A) and after (B) correction with a BME-IGRM matrix material. Scatter plots show the response curves representing rate of “ > 20 mg kg^−1^” or “ > 100 mg kg^−1^” responses before (C, D) and after (E, F) correction with BME-IGRM.

The results of the second study were classified as “gluten free”, “low gluten” or “contains gluten” from the values determined by each kit, based on the 20 mg kg^−1^ and 100 mg kg^−1^ legislative thresholds (Fig. 2). The measurement uncertainty was incorporated applying a decision rule chosen in accordance with Eurachem/CITAC (Eurachem/CITAC., 2007) recommendations to provide at least 97.5% probability that the gluten concentration in a sample is less than the threshold (i.e. a low risk of false acceptance). Therefore, to be classified as “gluten free” or “low gluten” the reported value would have to be less than the threshold value minus two times the result’s standard measurement uncertainty. Out of 24 analysed samples only 4, which were found negative for gluten by all kits, had the same classification in all assays. For the remaining 20 samples, the highest degree of agreement was 5 identical classifications out of a possible 7 and this was observed for only 4 samples. A pairwise comparison of the qualitative agreement between kits (Table 2) revealed that only 6 out of 21 possible combinations produced identical statements, for more than 70% of the samples. Only one pair of kits (R-Biopharm and Transia) reached 100% agreement.

**Table 2:** Comparison of agreement between pairs of assays for results of the food samples (2nd study). Correlation: Pearson’s r values. Values of r that are above 0.8 are in bold. Quantitative and qualitative agreement: without any correction (No corr), after mathematical recalibration (Mat rec) and after correction with BME-IGRM material (BME-IGRM). Quantitative agreement: average between-assay RSD_ba_ in%. Qualitative agreement: number of identical statements produced by the two methods with regards to the gluten content (gluten-free, low gluten, contains gluten) per number of measured samples (in %).

Correlation[Pearson’s r]	AllerTek			Agraquant			Biomedal			Rbiopharm			Transia			Morinaga		
Neogen	**0.96**			0.72			0.73			0.61			**0.87**			0.30		
AllerTek				0.62			0.59			0.55			0.78			0.25		
Agraquant							0.77			**0.88**			**0.91**			0.66		
Biomedal										**0.81**			**0.95**			0.25		
Rbiopharm													**0.85**			**0.87**		
Transia																0.66		

Quantitative agreement RSD_ba_ [%]	*No corr*	*Mat rec*	*BME-IGRM*	*No corr*	*Mat rec*	*BME-IGRM*	*No corr*	*Mat rec*	*BME-IGRM*	*No corr*	*Mat rec*	*BME-IGRM*	*No corr*	*Mat rec*	*BME-IGRM*	*No corr*	*Mat rec*	*BME-IGRM*

Neogen	120	11	13	57	31	43	79	38	62	98	39	61	108	35	49	118	40	61
AllerTek				96	34	44	69	33	60	57	37	58	49	32	50	54	38	57
Agraquant							48	28	48	80	29	34	91	22	31	110	32	36
Biomedal										46	25	31	57	22	64	87	33	41
Rbiopharm													22	20	39	55	27	29
Transia																48	26	44

Qualitative agreement [%]	*No corr*	*Mat rec*	*BME-IGRM*	*No corr*	*Mat rec*	*BME-IGRM*	*No corr*	*Mat rec*	*BME-IGRM*	*No corr*	*Mat rec*	*BME-IGRM*	*No corr*	*Mat rec*	*BME-IGRM*	*No corr*	*Mat rec*	*BME-IGRM*

Neogen	0	94	78	78	67	44	28	83	33	6	78	39	6	83	56	0	61	39
AllerTek				0	61	44	39	78	28	78	72	28	78	78	44	61	56	28
Agraquant							50	72	67	0	89	83	0	83	67	0	61	61
Biomedal										50	83	83	50	89	67	33	78	61
Rbiopharm													100	94	83	72	72	78
Transia																72	78	72

To examine the impact of these discrepancies on the level of consumer protection afforded by the results, we plotted the percentage of all kit results above the legislative thresholds versus the median concentration observed for each sample (Fig.2C). As the true value of gluten concentration in the samples from the second study was not known, the median of all results from each sample was considered the best estimate available. A logistic regression was used to provide a fit to the data. Based on the obtained response curve, an estimate of the reliability of the ELISA test kits’ results at any gluten concentration of interest can be determined (Magnusson & Ornemark, 2014).

The data in Fig.2C suggests that with the current gluten measurement system, as modelled by this study, the threshold of 20 mg kg^−1^ ensures that the gluten concentration in food products labelled as gluten-free would not exceed a level between 80 and 90 mg kg^−1^, independent of the test kit used. This is the concentration below which the current gluten measurement system becomes unreliable or agreement between different test kits becomes less likely. At 30 mg kg^−1^ the probability of being classified as gluten-free, within the current measurement system is nearly 40%. However, from the perspective of a food producer, to obtain a 95% probability that the product will conform to the current requirements for gluten-free labelling by any of the test kits, the actual gluten content in the sample has to be well below 5 mg kg^−1^.

The second legislative threshold of 100 mg kg^−1^ (Fig.2C) ensures consumer protection at the practical level of 210–220 mg kg^−1^. For samples below 210 mg kg^−1^, the responses provided by gluten measuring assays are unreliable and at the 100 mg kg^−1^ level the probability of an incorrect classification is 50% and still exceeds 20% at 150 mg kg^−1^. If a food producer wishes to have a 95% assurance that his product will not be classified in the EU as “contains gluten”, the actual concentration of gluten in a product needs to be less than 20 mg kg^−1^.

This directly translates into a risk model of consuming gluten-containing food when a dietary choice is made based on labelling information assured by the current measurement system. The above illustrates the current difficulties in providing protection to consumers based on the legislative levels using the current ELISA based measurement infrastructure.

#### 3.1.2 Assay performance – Boundaries for harmonisation

The achievable degree of equivalence between results from different test kits is very much dependent on the performance characteristics of each individual assay. The validation data provided by manufacturers for the repeatability and reproducibility of the kits used in this study are presented in Table 1, which also includes our assessment of the repeatability and intermediate precision as observed in this study. The relative standard deviation (RSD) of averages within (first study, spiked samples and cookies) and between (second study, food samples) ELISA plates for spiked and incurred samples was calculated. In addition, the within-plate variation of multiple absorbance readings for the same extracts was evaluated. This within-plate variation was low, 5% on average, for all the ELISA assays studied. The within-run RSD_wr_ (independent extracts of the same sample analysed on the same ELISA plate) was higher with an average value of 10% (7% to 14%). The average between-run RSD_br_ (independent extracts of the same sample analysed on two different ELISA plates from the same manufacturer’s batch) was 13% (7–20%). The experimental setup did not provide data on the reproducibility of individual kits. According to the reports from collaborative studies, it is expected to be in the range of 10% to 37%, however, data are not available for all the kits studied.

The precision attainable by individual kit assays sets the limit for possible harmonisation of gluten measurements. This can only be improved by kit producers by further optimization of their analytical protocols and manufacturing processes. In particular, the repeatability of extraction should be scrutinized, as the variation between extracts seems to constitute one of the most prominent components of within-kit variability. In our study, however, we focused on the aspects of the measurement procedure that can be challenged on a more global scale such as the use of universal calibrators, extraction procedure and the provision of matrix reference materials.

### 3.2 Evaluation of impact of uniform extraction procedure

All ELISA measurement protocols aim to quantify the total gluten content in a food sample, but full extraction of all gluten proteins is problematic due to differences in the solubility of the two main classes of proteins in gluten (Diaz-Amigo & Popping, 2013). The extraction protocols included in commercial ELISA test kits are not harmonized and comprise proprietary extraction buffers, of largely undisclosed composition and use diverse incubation times and temperatures (Table 1).

In this study, as inspired by the Japanese official method for allergen detection, a generic extraction procedure was applied (Akiyama et al., 2011). The vital wheat gluten was extracted with each kit’s extraction procedure as well as with the generic extraction procedure. The obtained extracts were consecutively diluted to cover each kit’s quantification range and measured by ELISA according to each kit’s protocol.

**Fig. 3:**
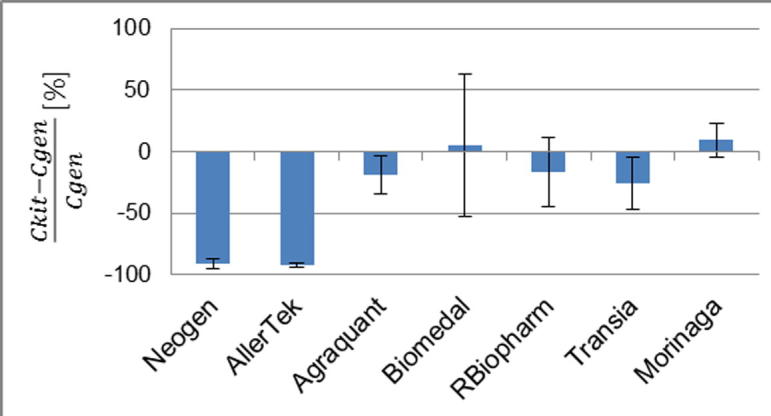
Comparison of efficiency of extraction with the kit-supplied and generic procedure. Relative differences between gluten concentration measured following the kit buffer extraction (Ckit) and gluten concentration measured following the generic buffer extraction (Cgen). Error bars represent the expanded uncertainty. The results were expressed as the averages of the relative differences between gluten content measured following the kit buffer extraction and gluten content measured following the generic buffer extraction.

The results presented in Fig. 3 show that for most of the kits the efficiency of extraction with the kits’ buffers is roughly equal to the efficiency of extraction with the generic buffer, within the limits of the observed measurement uncertainty. For two kits (AllerTek and Neogen) the generic buffer extracted approximately 10 times more gluten than the respective proprietary buffers. These are the two kits which produced the most extreme gluten concentration values in our first and second studies. However, these two kits produced values which were at the opposite ends of the observed range of results. Furthermore, the group of kits that showed similar extraction efficiency did not produce comparable results for gluten content. The comparison reveals that differences in efficiencies of extraction by themselves do not explain the observed differences in the reported results for gluten content. Therefore, the introduction of a harmonized extraction protocol, in isolation, would not immediately improve the degree of equivalence between results of gluten measurements. During the development of an ELISA assay there might be a number of correction factors introduced by a manufacturer to compensate for issues such as non-quantitative extraction or antibody specificity.

#### 3.2.1 Evaluation of the impact of calibration procedures

The gluten ELISA test kits currently on the market use different calibrators ranging from extracts of vital wheat gluten or gliadin to a processed matrix material (Table 1). There is often no detailed information provided with regards to the origin of the calibrators, their varietal composition or value assignment procedure. A number of test kits claim traceability of measurement results to a material called WGPAT gliadin (van Eckert et al., 2006). In many cases the calibration protocol includes a correction factor which defines how a calibrator relates to total gluten in a sample.

The calibration curves, as generated using kit supplied calibrators and measurement procedures, were examined. All resulting plots showed increasing curvature at higher concentrations. For sample extracts with high gluten concentrations but still within the range of quantification stated by the manufacturer, a small variation in measured absorbance has a large impact on the determined concentration. This effect, combined with the estimation of the uncertainty for the polynomial regression, resulted in a large measurement uncertainty estimate for gluten concentrations that were determined from the upper portion of the polynomial curve. For example, the uncertainty contribution from the fitting of the polynomial regression model calculated for a concentration at ¼ of the calibration range was between 2% and 8%, this increased to between 4% and 12% when calculated at ¾ of the calibration range.

#### 3.2.2 Assessment of effect of the universal calibrator

To evaluate if the introduction of a uniform calibration procedure would improve the comparability of measurement results from different ELISA kits, the use of an industrial extract of vital wheat gluten as a universal calibrator was investigated. The gluten concentration value was assigned to the universal calibrator based on results of measurements of total protein content. The calibrator was extracted with each kit’s extraction procedure and then consecutively diluted to cover each kit’s quantification range and measured in accordance with each kit’s protocol. The determined gluten mass fractions (mg kg^−1^) were plotted against the assigned values and a linear regression was used to provide a fit to the data. The resulting regression coefficients were used to correct the data set obtained in the first study.

The corrected results were treated according to ISO 5725-2. The complete range of corrected measurement results was from 3 to 46 mg kg^−1^ for samples spiked/incurred with 20 mg kg^−1^ of gluten and from 16 to 215 mg kg^−1^ for samples spiked/incurred at the 100 mg kg^−1^ level. Contrary to the uncorrected data, the statistical analysis did not identify an individual test kit that consistently produced outlying results. The average repeatability and reproducibility standard deviations were 17% and 53% at 20 mg kg^−1^ and 8% and 47% at the 100 mg kg^−1^ level, respectively. The comparison of precision statistics obtained for corrected and uncorrected data sets showed that the effect of the introduction of the universal calibrator was similar to the effect of removal of results from the kit identified as an outlier in the assessment of the uncorrected data (Fig.1C). This suggests that the most prominent component of the variation of results observed in the first study was largely the biased calibration procedure of one test kit (AllerTek). However, there is an unknown source of bias present that was not reduced by the use of harmonized calibration procedures and accounts for the limited level of precision observed in the corrected data set.

Analysis of corrected data according to the International Standard 13528 showed little improvement in the overall performance statistics compared to the uncorrected results. The proportion of results with a satisfactory performance score was 77% compared to 70% prior to correction. Correction induced a shift in distribution of satisfactory performance between the kits (Fig. 1). The kit which scored unsatisfactory performance scores in all cases before correction (AllerTek), scored satisfactory performance for 50% of samples after correction. However, the negative bias observed before correction for another kit (Neogen) increased as a result of correction. This confirms that harmonisation of the calibration procedure reduced the bias that could be attributed only to the calibration procedure itself and that other significant, so far unidentified, sources of bias still persists.

#### 3.2.3 Assessment of the effect of the candidate matrix reference material BME-IGRM

The question remains whether a pure gluten extract was sufficiently fit-for-purpose to be used for the correction of results of measurements in matrix samples. To address this issue the suitability of a matrix material BME-IGRM, (Bugyi et al., 2012) consisting of baked cookies incurred with the WGPAT gliadin at the levels corresponding to 0, 20 and 100 mg kg^−1^ gluten, was evaluated. BME-IGRM was measured along with food samples during the second study. The measurement results obtained from this reference material were plotted against the incurred concentrations. The coefficients of the fitted regression lines were used to correct the values obtained for the food samples.

The corrected results were treated according to ISO 5725-2. For the food samples, no individual test kit was identified as providing discrepant results. The average repeatability standard deviation was 19% (ranging from 5% to 55%) and average reproducibility standard deviation was 57% (25–105%). Analysis according to the ISO 13528 yielded 70% satisfactory performance expressed as z scores.

In comparison to the results obtained from the uncorrected data set, there was a reduction in the observed reproducibility standard deviation (from 76% to 57%) and a reduction in the number of non-satisfactory performance z scores (from 57% to 30%). Although correction helped to improve the performance statistics for some test kits (Agraquant, Neogen, Morinaga), it didn’t bring any substantial change for the others. For kits where improvements were not observed, the correction modified the pattern of distribution of unsatisfactory results between the kits and between the samples (Fig.1D). Furthermore, the negative or positive biases became less evident and more scattered and the results that were not recognised as biased before correction received non-satisfactory performance scores afterwards.

The impact of normalising the results of the second study to the values obtained for the BME-IGRM was evaluated by assigning each sample as “gluten-free”, “low gluten”, or “contains gluten” (Fig.2B). Only 5 out of 21 samples resulted in the same classification by all test kits after correction. Three of these were found to contain no detectable gluten. The pairwise comparison of this qualitative agreement between kits, in relation to the uncorrected data, revealed improvements for some pairs of kits. However, for others the agreement became even poorer. The most remarkable increase in agreement was observed for a combination of AllerTek and Neogen (from 0% to 78%) and the combination of Agraquant with R-Biopharm, Transia and Morinaga (from 0% to 83%, 67% and 61% respectively). However, overall there was no change in the number of kits resulting in identical statements for greater than 70% of the samples. Not a single pair of kits reached 100% agreement after correction compared with 1 pair before correction.

The inspection of the curves plotted for qualitative responses observed after correction for BME-IGRM at each legislative threshold (Fig.2D) revealed some improvement of the reliability of the gluten measurement system as approximated by this experimental design. As a consequence of correction, the gluten concentration at which there is <5% probability of false classification as “gluten free” decreased from 80–90 mg kg^−1^ to 30–40 mg kg^−1^, and from 210–220 mg kg^−1^ to 150–160 mg kg^−1^ for “low gluten”.

### 3.3 Did the reference materials improve the comparability of results?

#### 3.3.1 Correlation analysis

To gain more insight into how the results from different ELISA kits are inter-related, a correlation analysis of the results obtained in the second study was performed. The scatter plots were used as graphical indicators of the association between the values generated by each pair of kits (data not shown). The Pearson’s product-moment correlation coefficient (r) was used to quantify the strength of the hypothesized relationship. A cut-off value of 0.8 was chosen to discriminate between meaningful and meaningless correlation. This cut-off was chosen because at correlation coefficients below 0.8 the scatter of points around the correlation line and confidence intervals estimated for Pearson’s r values at 95% probability, were so broad that the association between results was no longer significant.

The correlation coefficients for all 21 assay comparisons ranged from 0.25 to 0.96, indicating no correlation to a high degree of correlation between measurement results (Table 2). For 8 assay pairs, the value of r was above 0.8. These results highlight a group of four kits that were correlated (Agraquant, Biomedal, RBiopharm and Transia) and three pairs (Neogen-AllerTek, R-Biopharm-Morinaga, Transia-Neogen) that also showed a high degree of correlation.

A visual inspection of plots revealed that there were individual samples that behaved differently from most other samples. These outlying samples were excluded and the Pearson’s correlation coefficients were recalculated. The calculations were repeated to include only samples of similar matrix composition. In both cases, the resulting r and confidence interval values led to the same conclusions with respect to the significance of the calculated correlations.

The poor correlation between results generated by different assays makes it impossible for gluten measurements to achieve full comparability by using a reference material for calibration alone. To assess the degree of agreement potentially achievable, by using a reference material, a mathematical recalibration of the assays was performed. A Passing-Bablok regression line was fitted to the data for each individual assay from the second study versus all assay medians and the regression parameters were used to correct the results (Table 2).

Before mathematical recalibration, the average between-assay RSD_ba_ was 98% for all assays, this was reduced to 39% after applying the correction factors. The same operation was applied separately to groups of assays for which the correlation factors were higher than 0.8. This resulted in a reduction of average RSD_ba_ from 120% to 11% for the comparison of Allertek and Neogen, from 54% to 28% for R-Biopharm and Morinaga, and from 63% to 28% for Agraquant, Biomedal, RBiopharm and Transia.

Although this approach is very basic, these numbers provide an idea of the best case scenario that can be achieved with the current measurement system and what could be achieved if all the assays were correlated.

The pattern of distribution of meaningful correlations is largely reflected in how the use of the BME-IGRM reference material influenced the degree of agreement between pairs of assays. After recalibration with BME-IGRM, the most remarkable increase in qualitative agreement was observed for the combinations of assays with the highest Pearson’s r values (Table 2): from 0% to 78% for the pair Neogen-Allertek (r = 0.96), from 0% to 83% for RBiopharm-Agraquant (r = 0.88) and from 0% to 67% for Agraquant-Transia (r = 0.91). In the absence of meaningful correlation, the recalibration brought little or no improvement of agreement e.g. from 28% to 33% for Neogen-Biomedal (r = 0.73) or decrease from 78% to 28% for AllerTek-RBiopharm (r = 0.55).

#### 3.3.2 Commutability

There were some pairs of assays that had high Pearson’s r values but correction with BME-IGRM material did not improve the comparability of their results (e.g. for a combination of Biomedal and Transia, the r value was 0.95 but the degree of qualitative agreement changed from 50% to only 67% after correction). This is attributed to the relationship between the values measured for BME-IGRM and the test samples and the concept of commutability. The reference material is commutable when its behaviour towards a given measurement procedure is equivalent to the behaviour observed in routine test samples (ISO, 2009). In this study, routine test samples were represented by the real food samples included in the second study.

**Fig. 4:**
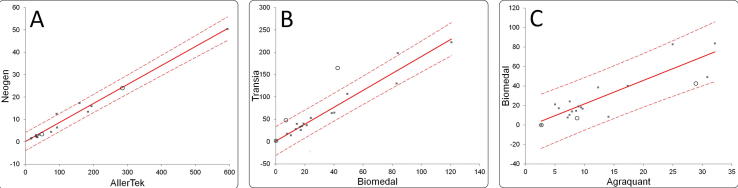
Examples of results for assessing the commutability of the BME-IGRM material for: A) where the material is commutable; B) where the material is non-commutable; and C) where the assessment is ambiguous due to a large spread of results around the regression line. Results for food samples are shown as black squares, results for BME-IGRM are shown as empty circles. Solid lines represent the regression line plotted for the food samples, dashed lines represent the 95% prediction interval.

As the full commutability study of incurred BME-IGRM material is not the primary subject of this paper, a basic approach for its assessment was adopted. A simple regression analysis was used to establish the relationship between the results obtained with each pair of assays and a 95% prediction interval was calculated, as implemented in Analyse-It, to describe the distribution of the ratio of results expected for the routine samples. Results, where the standardised residual was greater than three, were excluded as outliers and a new linear regression performed. The material was deemed commutable if the result ratio obtained for a reference material using the two methods was consistent with the prediction interval (ISO, 2009) (Fig. 4). The commutability of the BME-IGRM material was verified for the 7 pairs of assays for which the Pearson’s r value was higher than 0.8 and where the scatter of points on the correlation plots was not too large to calculate the meaningful prediction intervals. For 5 assay combinations, the commutability of the BME-IGRM material was acceptable. Whereas for 2 pairs of assays, the BME-IGRM material showed remarkable non-commutability (Transia-Biomedal, Transia-RBiopharm). The lack of commutability of BME-IGRM for these two combinations of methods correlates with a lack of improvement of agreement between the results of these methods, after the correction with this material. A similar picture is reproduced in the configuration of points in the Youden plots before and after correction with BME-IGRM (Fig.1D). The ellipses representing results generated by individual assays overlap more closely for the groups of correlated assays after the recalibration with a commutable material whereas the ellipse representing results of Transia is even more separated from the ellipses of R-Biopharm and Biomedal after correction. The lack of commutability of BME-IGRM between some assay pairs also explains the differences in agreement achievable globally for all assays, as estimated by average between-assay RSD_ba_ for the second study after mathematical recalibration (39%) and the degree of agreement achieved after recalibration with BME-IGRM (RSD_ba_ 57%).

The concept of commutability was developed in the field of clinical testing where many routine methods are based on measurement of various sub-components of complex biomolecular species that are assumed to relate directly to the level of the clinically relevant molecule in a sample (ISO, 2009). Gluten measurements pose similar challenges because the measurand is a combination of complex molecules which can be present in different forms and in different proportions in a sample, depending on its varietal composition and degree of alterations arising from food processing or sample preparation. Such structural differences between samples with the same total gluten content may introduce a bias between results obtained with one measurement procedure relative to the results obtained with other procedures. Moreover, the sensitivity of a measurement procedure to interferences resulting from differences between the reference material matrix and sample matrices needs consideration. Samples routinely analysed for gluten content represent different food products and thus different matrices. The identification of a complete set of routine samples, in relation to which the commutability of a reference material is assessed, is problematic. Therefore, in the food testing field, it may be necessary to develop a series of reference materials representing different matrix types.

In this study, we observed that some samples were outliers in many pairwise assay comparisons and showed exceptionally high between-kit variability in their results. One of these materials was a meat sample which falls in the 4th sector of the AOAC food matrix triangle which was different from most of the samples represented in this study. For the two other cases, the outlying samples were breakfast cereals which could be considered as representative of most of the samples studied. In the case of the meat sample, one could speculate that differences in responses may be attributed to matrix effects. However, for the two other samples, different responses were produced despite similar food matrices and may be due to differences in the composition and/or structure of the analyte. This may suggest that the basic assumption in food analysis that foods falling within the same sector of the AOAC food matrix triangle should be chemically similar and thus behave in a similar analytical manner (Wolf & Andrews, 1995), is questionable in the case of gluten analysis.

## 4 Conclusions

There were three main objectives of this study: to confirm previous observations that the measured gluten content of the same sample can differ greatly on different ELISA platforms; to understand the effect this spread has on the enforcement of current EU legislation; to observe the effects that standardisation would have on making gluten results comparable.

The observed discrepancies between kits in determining the gluten content were large, with the reproducibility (between-kit RSDs) ranging from 46% to 124%, depending on the sample. This was despite the precision of individual kits being below 20% and the fact that all results in this study were generated in one laboratory. The only samples that gave rise to the same outcome, from a food labelling perspective, were those that had no detectable gluten present. None of the other twenty samples would have resulted in the same legislative outcome being suggested by all kits.

This study suggests that the current measurement system for gluten quantification cannot fully support the gluten-free claims as defined by legislative requirements. A qualitative response model revealed that there is a 50% probability that a food product deemed compliant at the 20 mg kg^−1^ threshold on the basis of measurements performed by commercial ELISA test kits may, in reality, contain up to 80–90 mg kg^−1^ of gluten. While measurements of gluten at the supplementary 100 mg kg^−1^ threshold for “low gluten” resulted in a 50% probability that the real gluten content was higher than 100 mg kg^−1^ and could even be as high as 210–220 mg kg^−1^.

The lack of correlation between pairs of test kits suggests that, not only do the kits target different gluten markers but, these markers may not be present in gluten at a constant ratio and/or the effect of food processing may alter these markers. The introduction of reference materials can do little to improve measurements where this lack of correlation is observed. Where correlation between pairs of test kits was proven to be significant, the inclusion of a reference material in the measurement system was seen to improve the comparability of results. However, these beneficial effects were not observed equally by all kits or even for all matrices on the same kit.

The measurement of gluten is proving to be a complex issue. The lack of comparable results could result in a potential detrimental exposure to those suffering from a gluten intolerance/allergy or an unnecessary product recall by food industries. Without agreement on a single common gluten marker, it is clear that the availability of a matrix reference material will not improve the situation for all kits and may even make it worse if used with some kits. Therefore, a greater need for consensus on acceptable robust markers and the conversion of the quantities of these markers to “gluten content” is required before conventional standardisation practices will have the desired effects.
